# Draft Genome Sequences of 12 Leuconostoc carnosum Strains Isolated from Cooked Ham Packaged in a Modified Atmosphere and from Fresh Sausages

**DOI:** 10.1128/MRA.01247-19

**Published:** 2020-01-09

**Authors:** Francesco Candeliere, Stefano Raimondi, Gloria Spampinato, Moon Yue Feng Tay, Alberto Amaretti, Joergen Schlundt, Maddalena Rossi

**Affiliations:** a Department of Life Sciences, University of Modena and Reggio Emilia, Modena, Italy; b BIOGEST—SITEIA, University of Modena and Reggio Emilia, Modena, Italy; c Nanyang Technological University Food Technology Centre (NAFTEC), Singapore; d School of Chemical and Biomedical Engineering, Nanyang Technological University, Singapore; Loyola University Chicago

## Abstract

Leuconostoc carnosum is a lactic acid bacterium that preferentially colonizes meat. In this work, we present the draft genome sequences of 12 Leuconostoc carnosum strains isolated from modified-atmosphere-packaged cooked ham and fresh sausages. Three strains harbor bacteriocin genes.

## ANNOUNCEMENT

Leuconostoc carnosum is a heterofermentative, catalase-negative lactic acid bacterium (LAB) comprising the major component of the microbiota in modified-atmosphere-packaged (MAP) cooked ham at the end of the shelf life, where it reaches a concentration of 10^7^ to 10^8^ CFU/g (1). After a few weeks of shelf life, a high dose of alive *L. carnosum* may be ingested when consuming MAP cooked ham (1). *L. carnosum* easily colonizes several other meat-based food matrices, as suggested by the species name (2–6). It has been associated with ham spoilage (7, 8), but some strains have been proposed as bioprotective starters (4, 9) due to their production of bacteriocins against Listeria monocytogenes (5, 6). When we started this project, a sole completed genome sequence was available in GenBank (*L. carnosum* JB16, accession number SAMN02603179), obtained from a strain isolated from kimchi (10). Twelve *L. carnosum* genomes have been sequenced to characterize this species, belonging to 9 strains isolated from MAP cooked ham and 3 from fresh sausages, according to Raimondi et al. (1, 2) (Table 1).

**TABLE 1:** Statistics of assembled genomes

GenBank accession no.	SRA accession no.	No. of raw reads	Strain	Source	Country of origin	Genome size (bp)	No. of contigs^*a*^	*N*_50_ (bp)	Coverage (×)^*b*^	G+C content (%)^*a*^	No. of CDS^*c*^	No. of rRNAs^*d*^	No. of tRNAs^*d*^	Completeness (%)^*e*^
VBXG01000000	SRR10389907	2,103,282	WC0318	Cooked ham	Italy	1,745,630	15	1,142,374	638	37.2	1,739	5	51	99.476
VBXF01000000	SRR10389906	1,718,930	WC0319	Cooked ham	Italy	1,700,071	11	1,137,580	521	37.1	1,679	5	49	99.476
VBXE01000000	SRR10389899	1,848,530	WC0320	Cooked ham	Poland	1,812,114	18	1,109,738	561	37.1	1,853	5	51	99.476
VBXD01000000	SRR10389898	1,753,950	WC0321	Cooked ham	France	1,804,293	40	1,106,035	532	37.1	1,854	5	52	99.476
VBXC01000000	SRR10389897	1,696,897	WC0322	Cooked ham	France	1,853,239	16	306,690	515	37	1,898	5	50	99.476
VBXB01000000	SRR10389896	1,847,670	WC0323	Cooked ham	Germany	1,773,698	23	256,026	560	37.2	1,802	5	52	99.476
VBXA01000000	SRR10389895	1,515,136	WC0324	Cooked ham	Germany	1,765,760	13	1,137,276	460	37.1	1,768	5	49	99.476
VBWY01000000	SRR10389893	1,654,963	WC0325	Cooked ham	Italy	1,830,248	27	229,291	502	37.2	1,881	5	52	99.476
VBWW01000000	SRR10389905	1,893,372	WC0326	Sausage	Italy	1,770,048	22	412,621	574	37.2	1,767	5	49	99.476
VBWV01000000	SRR10389904	1,872,273	WC0327	Sausage	Italy	1,769,683	20	412,621	568	37.2	1,768	5	49	99.476
VBWS01000000	SRR10389901	1,774,316	WC0328	Sausage	Italy	1,815,949	36	239,739	538	37.1	1,826	5	51	98.953
VBWR01000000	SRR10389900	1,498,696	WC0329	Cooked ham	France	1,650,966	14	256,123	455	37.2	1,644	5	52	99.476

a Determined using QUAST v5.0.2.

b Coverage has been calculated as (2 × 250 × number of reads)/reference genome length.

c Determined using the RAST server.

d Determined using Prokka v1.13, with default parameters.

e Determined using CheckM v1.0.8.

For whole-genome sequencing, each strain was cultivated in brain heart infusion broth (Becton, Dickinson, USA) at 30°C for 48 h under microaerophilic conditions. Biomass was collected by centrifugation for 5 min at 5,000 × *g.* The genomic DNA was extracted with a QIAamp DNA minikit (Qiagen GmbH, Germany). The concentration was determined with a Qubit fluorometer (Invitrogen, USA). The DNA samples were submitted to the Singapore Centre for Environmental Life Sciences Engineering (SCELSE), where they were tagged with Illumina TruSeq high-throughput (HT) DNA dual barcodes for library preparation and sequenced on an Illumina HiSeq 2500 instrument (USA). For each sample, 250-bp paired-end reads were obtained. The raw and postprocessed reads were checked for quality with FastQC v0.11.7 (11). Cutadapt v1.16 was used to trim Illumina adapters, and reads with a quality score lower than 20 (–overlap = 15, –minimum-length = 30, –quality-cutoff = 20) were removed (12). The trimmed reads were assembled with SPAdes v3.12 (–careful –cov-cutoff auto -k auto) (13), and the quality of the assemblies was evaluated using QUAST v5.0.2 (14). Trimming, quality checking, and assembly were performed on the Galaxy platform (https://usegalaxy.eu/) (15). The completeness of the assemblies was determined with CheckM v1.0.8 (16). The taxonomy was confirmed with KmerFinder on the CGE server (https://cge.cbs.dtu.dk/services/KmerFinder/) (17). The assembled genomes were ordered with Mauve v2.4.0 (18), using the sequence of *L. carnosum* JB16 as a reference. The draft genomes were annotated on the RAST server (19) and with Prokka v1.13 (20). BAGEL4 (21) was used to identify bacteriocin genes. The sequence data were deposited in GenBank under the BioProject accession number PRJNA542256. Table 1 shows the GenBank accession number, number of raw reads, assembly statistics, and features of the genomes. The average G+C content ( ± the standard deviation) was 37.14% ± 0.07%. An average of 1,790 coding sequences (CDS) per genome were annotated, with genome sizes ranging between 1,650,966 and 1,853,239 bp and an average completeness of 99.43%. According to *in silico* analysis, 3 out of 12 strains were identified as potential bacteriocin producers.

### Data availability.

The sequence data were deposited in GenBank under BioProject accession number PRJNA542256. Table 1 lists, for each sample, the GenBank accession number and the number of raw reads.
